# Effect of hyperbilirubinaemia on neurocognitive, renal, bone and cardiovascular markers in HIV infection treated with boosted protease inhibitors

**DOI:** 10.7448/IAS.17.4.19827

**Published:** 2014-11-02

**Authors:** Tristan J Barber, Graeme Moyle, Andrew Hill, Gurmit Jagjit Singh, Marta Boffito, Mark Nelson

**Affiliations:** St Stephen's Centre, Chelsea and Westminster Hospital, London, UK

## Abstract

**Introduction:**

Use of some protease inhibitors (PI) is associated with unconjugated hyperbilirubinaemia (HBR), due to inhibition of UGT1A1. As observed in Gilbert's syndrome, HBR may have antioxidant and anti-inflammatory effects. Inflammation may be relevant to neurocognitive (NC) impairment, cardiovascular, renal and bone co-morbidities in HIV infection. This study aimed to analyse correlations between antiretroviral associated HBR and NC impairment as well as renal, bone and cardiovascular parameters.

**Material and Methods:**

This cross-sectional study included 101 HIV-1-infected individuals stable (>6 months) on antiretroviral regimens including tenofovir/emtricitabine or abacavir/lamivudine plus a ritonavir-boosted PI. Patients with >grade 2 HBR were compared to patients with normal bilirubin on NC data collected using CogState. An overall composite score was calculated for each subject. Two-tail P-values were calculated using the Mann-Whitney U test. We measured the following parameters in all participants: Bone – Calcaneal Stiffness Index (CSI), blood bone markers, calculated FRAX score; CV – vascular endothelial function markers (iCAM, vCAM), lipid fractions and sub fractions (Total, HDL and LDL cholesterol, triglycerides, ApoB), Carotid Intimal Thickness (CIT), Pulse Wave Velocity (PWV), glucose and insulin for calculation of HOMA-IR, IL-6, d-dimer, uric acid, and hsCRP; Renal – urea and electrolytes (U&E), urinary protein/creatinine ratio (uPCR), urinary retinal binding protein (RBP)/creatinine ratio.

**Results:**

Forty-three participants had normal bilirubin (NBR) levels and 35 had high bilirubin (HBR; >2.5 times upper limit); the remaining 23 patients had intermediate bilirubin levels or violated the protocol. The mean age of participants was 48 years; 93% were male and 84% Caucasian. Mostly no significant differences were seen in any of the markers when comparing the NBR and HBR groups. Two component tests of the CogState were seen to be different – visual learning and memory (OCL) and working memory (ONB) (Table 1).

VCAM1 and FRAX scores were noted to be different between the two groups, but the latter had a worse outcome in the HBR group.

**Conclusions:**

The numbers seen here were not felt to be large enough to draw clear conclusions around clinical significance. In the context of overall cognitive screening, the individual test differences were also not felt to be clinically significant. Clearly there are some early signs here of differences that may be worth investigating further in a larger, prospective study.

**Table 1:** Summary of relevant neurocognitive, cardiovascular and bone parameters

		NBR (n=43)	HBR (n=35)	p
Visual Learning and Memory; OCL — acc	mean (sd)	0.94 (0.11)	0.98 (0.07)	0.05
Working Memory ONB — acc	mean (sd)	1.22 (0.23)	1.34 (0.13)	0.03
VCAM-1 (ng/mL)	Median (IQR)	416 (353–497)	377 (319–416)	0.03
FRAX score	Mean	3.8	5.5	
	S.D.	2.1	4.1	
	Median	2.9	5.5	
	IQR	2.5–5.2	2.6–6.5	0.017
At risk (FRAX>3%)		20/42 (47.6%)	22/32 (68.8%)	0.098 (NS)

